# Emerging Roles of the Membrane Potential: Action Beyond the Action Potential

**DOI:** 10.3389/fphys.2018.01661

**Published:** 2018-11-21

**Authors:** Lina Abdul Kadir, Michael Stacey, Richard Barrett-Jolley

**Affiliations:** ^1^Institute of Ageing and Chronic Disease, University of Liverpool, Liverpool, United Kingdom; ^2^Frank Reidy Research Center for Bioelectrics, Old Dominion University, Norfolk, VA, United States

**Keywords:** ion channels, membrane potential, resting membrane potential, proliferation, stem cells, cancer, neurons, blood pressure

## Abstract

Whilst the phenomenon of an electrical resting membrane potential (RMP) is a central tenet of biology, it is nearly always discussed as a phenomenon that facilitates the propagation of action potentials in excitable tissue, muscle, and nerve. However, as ion channel research shifts beyond these tissues, it became clear that the RMP is a feature of virtually all cells studied. The RMP is maintained by the cell’s compliment of ion channels. Transcriptome sequencing is increasingly revealing that equally rich compliments of ion channels exist in both excitable and non-excitable tissue. In this review, we discuss a range of critical roles that the RMP has in a variety of cell types beyond the action potential. Whereas most biologists would perceive that the RMP is *primarily* about excitability, the data show that in fact excitability is only a small part of it. Emerging evidence show that a dynamic membrane potential is critical for many other processes including cell cycle, cell-volume control, proliferation, muscle contraction (even in the absence of an action potential), and wound healing. Modulation of the RMP is therefore a potential target for many new drugs targeting a range of diseases and biological functions from cancer through to wound healing and is likely to be key to the development of successful stem cell therapies.

## Introduction

The fact that cells have a transmembrane potential has been known for over a 100 years, with earlier experiments by Hober (1905) establishing the observation, and Curtis and Cole (1942) and others demonstrating that it is maintained by the differential permeability of the plasma membrane to ions. This is termed the RMP and is typically in the range of −10 to −100 mV. The Nobel Prize break-through work by Hodgkin and Huxley (1952b) characterized in detail how rapid changes in membrane ion permeabilities lead to a regenerating action potential in nerves. This work was then refined and extended to describe the propagation of action potentials in other excitable tissues such as skeletal muscle and cardiac muscle (Neher and Sakmann, 1976). It rapidly entered dogma that the RMP was essentially a cocked-gun leaving excitable cells ready to fire depolarising action potentials. In nerve, clearly this serves the purpose of transmitting signals along their length from one part of the animal to another, whereas in skeletal and cardiac muscle it was hypothesized to spread the excitation signal throughout the tissue and was ultimately coupled to the elevation of intracellular Ca^2+^ and the excitation-contraction coupling. But what of *non-excitable* tissues? Most cells in an animal still have a dynamic membrane potential despite not having an action potential firing phenotype. Therefore, the role of the membrane potential is more enigmatic. Initial speculation could be that such non-excitable cell membrane potentials are an accident of evolution, however, detailed analysis of the literature shows that the membrane potential sub-serves a large range of essential biological functions (Table 1). In each case, relatively subtle differences in ion channel expression leave cells with quite distinct membrane potential properties; both in terms of level and potential for its modulation. The mechanisms, and ion channels controlling the RMP are vast and beyond the scope of this short review, so we focus instead on a range of distinct roles that the RMP plays across a selection of excitable and non-excitable cell types in a range of systems.

**Table 1:** Different functions and the cell types associated with these functions that are regulated by the RMP are shown.

Function	Cell type	Reference
Circadian rhythm	Neurones	Belle et al., 2009
	Fibroblast	Noguchi et al., 2012
Biological sensing	Neurones	Feetham et al., 2015b
Contractility	Vascular smooth muscle cells	Nelson and Quayle, 1995
	Myofibroblasts	Chilton et al., 2005
Hearing	Cochlea outer hair cells	Ashmore, 2008
Volume control	Chondrocytes	Lewis et al., 2011a
	Retinal Muller cells	Netti et al., 2017
Secretion	Pancreatic β cells	Aguilar-Bryan and Bryan, 1999; Lin et al., 2005
Proliferation	Vascular smooth muscle cells	Nelson and Quayle, 1995
	Stem cells	Messenger and Warner, 1979
	Cancer cells	Wonderlin et al., 1995
Cell cycle	T-cells	Amigorena et al., 1990
	B-cells	Neher and Sakmann, 1976
Cancer progression	Melanocytes	Chernet and Levin, 2013a,b; Blackiston et al., 2011
Migration and wound healing	Corneal epithelial cells	Reid and Zhao, 2014; Chifflet et al., 2005
	Skin keratinocytes	Nuccitelli, 2003
Pigmentation	Skin melanocytes	Bellono et al., 2013
	Uveal melanocytes	Hodgkin and Huxley, 1952b
	Retinal pigment epithelium (RPE)	Cone and Tongier, 1973

## Circadian Rhythm

Circadian rhythm is the term used to describe a range of biological processes that change in a daily 24-h cycle. One of the key master controllers is the master clock located in the SCN of the hypothalamus which coordinates the circadian pattern (Reppert and Weaver, 2002). The neuronal firing frequency changes with a daily cycle and thus leads to cyclic changes in the melatonin secretion from the pineal gland. Melatonin, in turn, modulates a range of biological processes (such as sleep) via interaction with the melatonin MT1/2 G-protein coupled receptors (Dubocovich, 2007). One of the fundamental signals mediating the circadian firing cycles of SCN neurones is change in the RMP. The mechanisms by which this occurs are counter intuitive to many people who would incorrectly see depolarisation of the RMP (when cells become less negative) as always excitatory and hyperpolarization of the RMP (when cells become more negative) as necessarily inhibitory. The earliest work on the RMP involved skeletal muscle and the squid giant axon since they are physically large enough to allow penetration with a recording electrode coupled to a Galvanometer adapted to measure small voltages (Hodgkin and Huxley, 1952b). In the early experiments, it became evident that small depolarisations led to the activation of action potentials. Therefore, intuitively small hyperpolarizations move cells further away from the trigger point and render cells less excitable (or inhibited). However, electrophysiology is very subtle, and the excitability of a nerve or muscle cell depends not only on the level of the membrane potential, but the overall conductance of the cell (the input resistance). Furthermore, depolarisation of cells does not always lead to activation, in some instances; it can leave cells less excitable; for example, the voltage-gated ion channels that ordinarily underlie the action potentials become inactivated and are no longer available for activation. This is how the firing frequency of neurones is regulated in the SCN. Under control of the *Per1* clock-gene, large and small Ca^2+^-activated K^+^ channel conductance is decreased in the afternoon, leading to a profound depolarisation of the RMP and a cessation of action potential firing in excitable cells (Belle et al., 2009).

Since many other cell types exhibit circadian cycling of clock gene expression, and consequent changes in cellular activity, it is intriguing to know if they too are associated with changes in the RMP. Fibroblasts are one such example of peripheral, non-excitable cells that display changes in RMP that follow a circadian cycle. It is not entirely clear why these cells do this, however, one hypothesis is that it is to adapt these cells to the small systemic changes in body temperature that occur throughout the day (Izumo et al., 2003). Whilst no direct RMP measurements have been made, ion channel blockers eliminate daily cycling of clock gene expression (Noguchi et al., 2012). The observation that the membrane potential of fibroblasts displays a circadian variation is consistent with the possibility that it plays an important role in non-excitable cells.

## Biological Sensing

Many cells have the constitutive ability to detect and respond to changes in their environment. In recent years, it has been clear that transient receptor potential (TRP) channels commonly underlie this behavior (Guilak et al., 1994; Clapham, 2003). In the case of neurones, our own studies have shown that neurones within the PVN respond to osmolality changes modulated by hypotonic saline which leads to the hyperpolarization of the membrane potential, which in turn reduces their firing frequency (Feetham et al., 2015b) and thus, ultimately control the blood pressure (Feetham et al., 2015a). In both of the systems above, and in other tissues including VSM (Nilius and Droogmans, 2001), a common mechanism appears to be a feedback loop, where Ca^2+^ increase activates Ca^2+^-activated K^+^ channels which hyperpolarize the plasma membrane and thus increase the driving force (voltage) for further Ca^2+^ entry (whereby the Driving Force is [G × (RMP – E_Ca_)] where G is conductance of the Ca^2+^ entry pathway and E_Ca_ is equilibrium potential for Ca^2+^ ions). This in turn increases the activation of Ca^2+^-activated K^+^ channels and enables the feedback loop to continue. We were able to simulate this numerically in the case of the PVN neurones (Feetham et al., 2018). We demonstrated that the Ca^2+^ elevation appears to be mediated by TRPV4 channels, and that the small-conductance Ca^2+^-activated K^+^ channels (SK channels) mediate a hyperpolarization that both increases intracellular Ca^2+^ and decreases their action potential firing frequency (Feetham et al., 2015b). Similar mechanisms are likely to be involved with the control of many other cell types, yet this phenomenon is best characterized in VSM (see below).

## Contractility

Despite many being non-excitable, smooth muscle cells are heavily dependent on their RMP for control of contraction. Some of the best-studied cells are vascular smooth muscle cells (VSMs) as they have considerable plasticity in their phenotype. They have the ability to change phenotype depending on their microenvironment from contractile cells to non-contractile cells and vice versa (Frid et al., 1997). VSMs function to drive the contraction of the vascular wall and regulate the luminal diameter and vascular tone. Unlike skeletal and cardiac muscle, whose contraction depends on “all or nothing” action potentials, VSMs contraction typically depends on the RMP changes through the K^+^ channel feedback loop mechanism to contract. The activation of K^+^ ion channels for example, the K_2P_, causes the efflux of K^+^ ions and hyperpolarization which brings the membrane potential to a value more negative than the threshold for activation of Ca^2+^ channels. This results in decreased Ca^2+^ influx, leading to the relaxation of the VSM and hence vasodilation (Nelson and Quayle, 1995). On the other hand, the inhibition of K_2P_ channels leads to the depolarisation of the membrane potential and the contraction of smooth muscle cells (Nelson and Quayle, 1995). Other non-excitable cell types that contract due to changes in the RMP include myofibroblasts and ventricular fibroblasts (Chilton et al., 2005). The myocardium is composed of different cell types. Cardiac myocytes occupy only a third of the cells in the cardiac myocardium; the remaining non-myocyte cells are mainly endothelial cells, VSMs and fibroblasts (Brilla et al., 1995). In myocardial injury, an immune response is generated, which results in the recruitment of fibroblasts which proliferate and transform into myofibroblasts (Frangogiannis et al., 2002; Tomasek et al., 2002). These myofibroblasts show similar characteristics to fibroblasts as they also proliferate and migrate and synthesize various autocrine and/or paracrine factors (Sun and Weber, 2003). Increasing the extracellular K^+^ ion concentration of ventricular fibroblasts leads to increase in the K^+^ channel activity of inwardly rectifying channels (K_ir_). This results in the depolarisation of the membrane potential and enhances the contractility of the fibroblasts (Chilton et al., 2005). The role of the membrane potential in contraction is crucial. One such example mentioned above is its modulation of the contraction of VSMs, since failure of the VSMs to contract can lead to detrimental effects in the blood flow to the heart. Perhaps, this explains why there are many ion channels that maintain the RMP as the heart’s change in metabolic demand needs to be met promptly and accurately.

## Hearing and the Mammalian Cochlear

**FIGURE 1:**
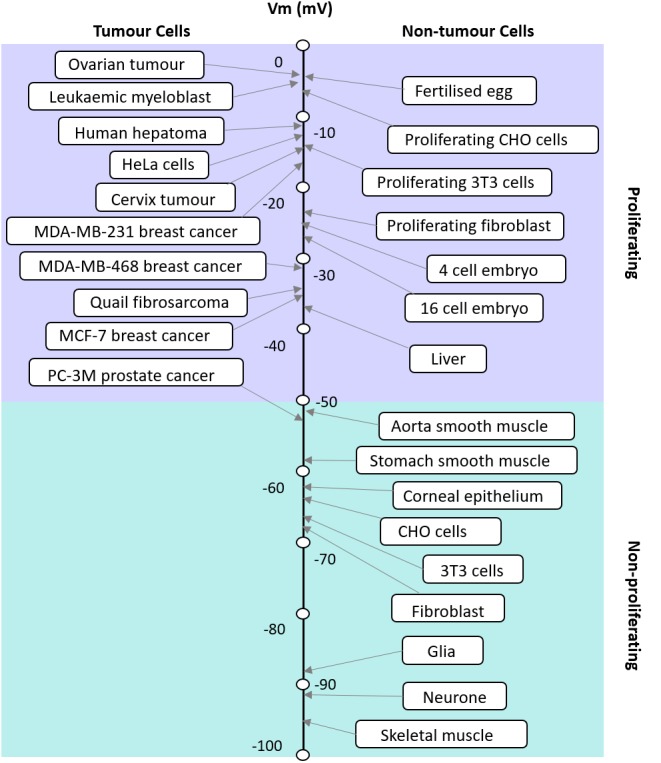
Apparent correlation between the RMP and proliferative potential of cells. The RMP of tumor and non-tumor cells and their proliferation potential are shown. Modified with permission from the copyright holders Yang and Brackenbury (2013).

The electromechanics of the cochlea are driven by the hair cell RMP (reviewed by Ashmore, 2008). The cochlea outer hair cells play a key role in sound amplification and fine frequency selectivity that works via two interrelated mechanisms: somatic electromotility (Brownell, 2017) and the active hair bundle (Harland et al., 2015). The cochlea of the mammalian inner ear is filled with two different extracellular solutions, the perilymph and endolymph, separated from each other by hair cells. The perilymph solution is composed of a “typical” interstitial fluid of 5 mM K^+^ and 150 mM Na^+^. Conversely, the endolymph solution is an atypical extracellular solution, composed of high K^+^ and low Na^+^ (150 mM K^+^ and 5 mM Na^+^) as well as 20 μM Ca^2+^ (Nin et al., 2016) as shown in Figure 1, and conveys an endocochlear potential of +80 mV. The outer hair cells expose cilia from their apical surface to the endolymph; this creates a very large electrochemical driving force for K^+^ entry and allows a relatively small mechanical (acoustic) activation of the KCNQ1/KCNE1 K^+^ channels to carry a massive *entry of K^+^ ions into* the hair cells, which in turn depolarizes the cell membrane. Influx of Ca^2+^ ions simultaneously occurs and contributes to establishing the dynamic ranges of hearing. The positive endocochlear potential, coupled to the hair cell RMP of ∼−45 mV contributes to finely tuned hearing profiles, and results in deafness when lost. This is the case with certain forms of albino associated congenital deafness, where there is an absence of the KCNJ10 expressing melanocytes (synonymous with intermediate cells) in the stria vascularis. This leads to a loss of the high endocochlear K^+^ concentration and an inadequate ionic driving force for K^+^ entry (Ryugo and Menotti-Raymond, 2012).

**FIGURE 2:**
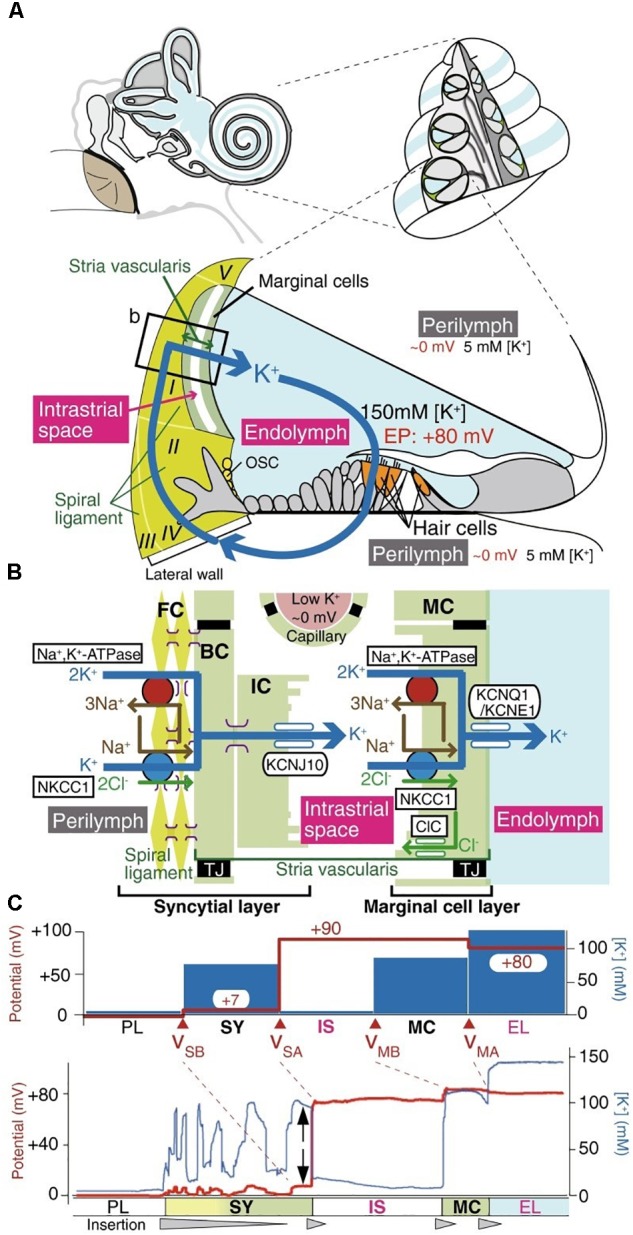
The electrochemical profile of the cochlea. **(A)** The structure of the human ear and a cross-section of the cochlea are illustrated in the upper panel. The tissue and cellular composition of the cochlea are illustrated in the lower panel; this shows the electrochemical properties of the endolymph and perilymph and the possible circulation of K^+^ ions between them. The boxed region in the lower panel is shown in **(B)**. **(B)** Structural and cellular composition of the lateral wall of the cochlea depicting key ion channels and transporters that are thought to maintain the unidirectional K^+^ transport across the lateral wall and the endocochlear potential (EP) they produce are shown. **(C)** Membrane potential and [K^+^] of the lateral wall under physiological conditions. The top panel displays the membrane potential and [K^+^] averaged from multiple measurements. The membrane potential across the basolateral and apical surfaces of the syncytial layer, vSB and vSA, respectively, and the basolateral and apical surfaces of the marginal cell layer, vMB and vMA, respectively, are shown. The lower panel shows a representative trace of the measurement of the membrane potential and the [K^+^] in the cochlea of a live guinea pig. [Reproduced and modified with permission from the copyright holders the National Academy of Sciences (Nin et al., 2008) and Springer-Verlag Berlin Heidelberg (Yoshida et al., 2016)].

In order to maintain the endocochlear potential, a K^+^ circuit is present; K^+^ entering the hair cell at the apical end is transported through the cell and exits into the perilymph. From here K^+^ is unidirectionally transported through the syncytial layer and into the Intrastrial Fluid (IF) before being actively transported by cells of the marginal layer back into the endolymph. The positive RMP of the syncytial layer (0 to ∼ +5 mV) (Nin et al., 2016) is critical for harvesting the K^+^ diffusion potential on the apical surface to set the intrastrial space potential and endocochlear potential to a highly positive value, which is essential for hearing (Figure 2). The syncytial basolateral surface is mainly provided by fibrocytes in the spiral ligament. In spite of the physiological importance of fibrocytes, the machinery underlying the establishment of this unique RMP have not yet been fully characterized. Currently, it is believed that changes in the membrane potential cause the transfer of an electrical charge inside the cell membrane resulting in conformational changes in prestin, a member of the SLC26 anion exchanger family, which induces conformational rearrangements in response to intracellular Cl^−^ concentrations and leads to morphological changes to the cell length. The complexities of the cochlea provides an excellent example of different yet distinct roles of the RMP in a functioning non-excitable tissue.

## Volume Control

In all cells, maintenance of cell volume is essential for survival. The membrane potential is likely to be a key regulator of this process in many cells, although, to date this has only been demonstrated explicitly in a few cell types including: cardiomyocytes, retinal Muller cells, and chondrocytes (Lewis et al., 2011a; Cha and Noma, 2012; Fernández et al., 2013). Active control of cell volume is especially true of chondrocytes, since they exist in an environment with constantly changing osmolality and compressive loads (Wilkins et al., 2000). The membrane potential feeds into the cell volume control mechanism by changing the “driving force” for ionic fluxes. In this context, the ion channels, especially those selective for K^+^, are behaving as “osmolyte” channels (Kerrigan and Hall, 2000) rather than simply maintaining the RMP as expected from dogma. We have demonstrated that in chondrocytes the maintenance of the RMP is heavily dependent on TRP channels, with TRPV5 being a key player (Lewis et al., 2011a), although there are still clear species-dependent contributions from other K^+^ channels. For example, the voltage-gated KV1.6 (deriving from the KCNA6 gene) was found to be a strong contributor, by a steady-state activation that remained 5% open even at −90 mV (Clark et al., 2010). Further work by the same group revealed that a major additional contribution to the RMP of human articular chondrocytes was provided by the TASK-2 two-pore domain K^+^ channel conferring, in particular, pH sensitivity (Clark et al., 2011). Our own examination of the chondrocyte RMP over many years across different species indicates that it is less negative than *published* for most other cell types. We proposed that the diverse compliment of chondrocyte ion channels in both articular (Barrett-Jolley et al., 2010) and costal cartilage (Asmar et al., 2016) contributes to this relatively positive RMP in chondrocytes and facilitates volume control as an adaptation to the extreme osmotic challenges these cells routinely face (Lewis et al., 2011b). Our data support this as they show that at negative membrane potentials chondrocytes appear to be unable to decrease their volume when exposed to higher osmotic potential solutions, i.e., cell shrinkage was slower or non-existent (Lewis et al., 2011a). This mechanism seems closely paralleled to that reported more recently for Muller cells (Fernández et al., 2013; Netti et al., 2017). These are a type of glial cell that supports the local homeostasis of the retina. Functionally they are thought to be quite similar to astrocytes found elsewhere in the CNS and this implies wider importance of the RMP/cell volume links throughout the CNS. Fernández et al. (2013) used potentiometric dyes rather than sharp electrodes, but were able to model a close relationship between the RMP and regulatory volume decrease in terms of both magnitude and kinetics of response. Further studies by the same group (Netti et al., 2017) show that TRPV4 is a key player in this process, again functionally coupling to Ca^2+^-activated K^+^ channels. This data indicates that the optimal RMP is unique to different types of cells depending on their function and environment; hence its maintenance is crucial for cell survival.

## Secretion

One of the most extravagantly studied secretory mechanisms is the pancreatic β-cell insulin secretion system. This system has membrane potential level control of secretion and is perhaps a model of secretion that is more widely known. β-cells express ATP-sensitive K^+^ (K_ATP_) channels; that is, K_ir_ that close in response to elevation of intracellular ATP (Aguilar-Bryan and Bryan, 1999). These K_ATP_ channels play a key role in the glucose-stimulated insulin release in pancreatic β-cells (Ashcroft et al., 1984; Ashcroft and Rorsman, 1990). When the blood glucose levels rise, there is an increase in the ATP concentration and a decrease in the ADP concentration which causes the K_ATP_ channels to close and the cell’s membrane to become depolarised leading to Ca^2+^ influx and therefore insulin release. On the other hand, when the blood glucose levels fall, ATP concentration decrease leading to the opening of the K_ATP_ channels, membrane hyperpolarization and the termination of insulin secretion. The route of Ca^2+^ influx is not entirely clear, but several Ca^2+^ channel α-subunits have been identified in pancreatic β-cells including CaV1.2, CaV1.3, CaV2.1, CaV2.2, CaV2.3, and CaV3.1, have been identified in pancreatic beta-cells and mutations of CaV1.3 and CaV2.1 channels are seen in subgroups of diabetic patients (Yang and Berggren, 2006). The K_ATP_ channels can also regulate the insulin release through their interaction with phosphoinositides in particular with PIP_2_ which stimulates K_ATP_ channels by decreasing their sensitivity to ATP, causing the cells to become more hyperpolarized and not secrete insulin properly when glucose levels are high (Lin et al., 2005). This highlights the importance of the RMP as mutations in the ion channels that contribute to the RMP can have detrimental effects that lead to disease.

## Proliferation

The RMP in excitable and non-excitable cells usually varies in a wide range from −10 to −100 mV. It has been postulated that where cells fall on this scale corresponds to their proliferative potential (Figure 1; Binggeli and Weinstein, 1986). For example, cells that have membrane potentials that are hyperpolarized tend to be quiescent and do not usually undergo mitosis; whereas cells that have depolarised membrane potentials tend to be proliferative and usually mitotically active (Cone, 1971). However, this is not always the case as each cell type is different and expresses different ion channels. The membrane potential is highly correlated with mitosis, DNA synthesis, cell cycle progression and overall proliferation in general (Cone, 1971; Binggeli and Weinstein, 1986). The relationship between the RMP and cells’ proliferation levels could be taken advantage of, to manipulate the cells’ mitosis and increase their proliferation when desired (Cone, 1971). Several studies have shown that changing the membrane potential of a particular cell can drive changes in the cell’s proliferation levels. For instance, it has been shown that the hyperpolarization of the Chinese hamster ovary (CHO) cells to −70 mV leads to mitotic arrest and this effect was shown to be reversible when the membrane potential was depolarised back to the normal RMP of −10 mV (Cone and Tongier, 1973). This effect seems to be the opposite of what is known in other cell types. Membrane potential control of proliferation is a complex relationship because the membrane potential relies on the activity of many ion channels. Cone and Tongier (1973) were not able to address the nature of the specific ion channels involved, but hypothesized that it would be Hodgkin Huxley (Hodgkin and Huxley, 1952a) like voltage-gated Na^+^ and K^+^ channels. Further work later explores a range of voltage-gated ion channels involved in this process (Rao et al., 2015), see “cell cycle” below. However, it is also important to note that the downstream events are different for every cell type and can be as a result of voltage or the flow of certain ions across the membrane.

Many of the studies on the relationship between the membrane potential and proliferation have been carried out on K^+^ channels and it has been shown that the inhibition of the K^+^ currents results in the inhibition of proliferation in many cell types such as lymphocytes, PBMCs, Schwann cells, astrocytes, oligodendrocytes, and different types of cancer cells (Wonderlin and Strobl, 1996; MacFarlane and Sontheimer, 2000). Additionally, in some cell types the inhibition of K^+^ currents results in the activation of proliferation. The identity of the specific potassium channels involved seems to be very cell type specific and indeed both Ca^2+^ activated K^+^ channels (KCNMA) (Garcia et al., 1991) and KV1.3 (KCNA3) channels (Garcia-Calvo et al., 1993) have been separately identified on the basis of toxin inhibition (charybdotoxin and margatoxin, respectively). Furthermore, it was shown that cells that are post-mitotic such as those in the CNS can be coaxed back to enter the cell cycle after sustained depolarisation. Depolarising astrocytes with ouabain causes their increased proliferation and DNA synthesis (MacFarlane and Sontheimer, 2000).

Contractility of VSMs, as previously mentioned, is regulated by the membrane potential; however, these cells can switch phenotype during injury or development (Frid et al., 1997). The different phenotypes have different ion channel expressions. Interestingly, there is a switch in the type of ion channels that are expressed in each phenotype which contribute to the regulation of the membrane potential and hence the contractility of the VSMs phenotype. For example, serum induced proliferation (a common VSM tissue culture technique) leads to elevations of the canonical transient potential channel (TRPC1) and elevated resting Ca^2+^ concentration (Stenmark et al., 2006). In another example, mitogen-driven proliferation in immune cells, which is mediated by K^+^ channels is also regulate by the membrane potential (Deutsch et al., 1986). Furthermore, numerous studies show that cancer cell proliferation is regulated by different ion channel modulators implying a role for the RMP. As well as that, the RMP has also been shown to regulate neuronal differentiation (Messenger and Warner, 1979). Recent data have shown that the RMP can also have an effect on the migration of cells where K^+^ channels (KCNQ1) and Na^+^ channels (NaV) were found to play a role in the migration of several stem-cell like cell types (Djamgoz et al., 2001; Morokuma et al., 2008). Additionally, the membrane potential also regulates proliferation through the modulation of the cell cycle. For example, the MCF-7 human breast cancer cell proliferation has been shown to require a characteristic RMP hyperpolarization during the G0/G1 phase of the cell cycle [46]. Regulation of the cell cycle through the RMP is further discussed below.

## Cell Cycle

As discussed earlier, the membrane potential can regulate proliferation levels within cells through regulating the cell cycle progression. A hyperpolarized membrane potential inhibits mitosis as it blocks quiescent cells in the G1 phase of the cell cycle from entering the S phase and hence blocks the DNA synthesis. It is hypothesized that voltage-gated Na^+^ and/or Ca^2+^ channels open to depolarise cells and promote transition from G0/G1 to the S-phase. Whereas opening of K^+^ voltage-gated channels (such as KV11.1, the human ortholog also known as hERG or KCNH2) and closure of Na^+^/Ca^2+^ channels during the S-phase tend to re-polarize the cell and lead back to G0/G1 (Rao et al., 2015). It is postulated that there may be a threshold RMP level that cells need to overcome in order to drive DNA synthesis in cells. For example the expression of certain ion channels in proliferating astrocytes can be upregulated or downregulated depending on the RMP levels of the cells and the cell cycle stage that they are in. A cell cycle arrest in the G1/G0 phase of proliferating astrocytes induces a premature upregulation of an inwardly rectifying K^+^ channel (K_ir_) that results in the hyperpolarization of the membrane potential. Conversely, a cell cycle arrest in the S phase of the cell cycle causes a downregulation of an outwardly rectifying K^+^ channel (K_ir_) and hence the depolarisation of the RMP (MacFarlane and Sontheimer, 2000). This indicates that there seems to be a G1 to S phase transition checkpoint that is regulated by the membrane potential. In addition, the inhibition of ion channels that modulate the membrane potential, such as for example K^+^ channels, causes the inhibition of progression of cells onto the next stage of the cell cycle. The inhibition of K^+^ ion channels in non-β lymphocytes and cytolytic T lymphocytes can cause the inhibition of these cells’ progression through the G1 phase of the cell cycle and hence inhibits their activation (Amigorena et al., 1990). This membrane potential control of the cell cycle can be utilized as a mechanism to inhibit cancer cell proliferation, making it a potential target for future treatments.

## Cancer Progression

Since cell cycle and cell proliferation are strongly influenced by the RMP, it is not surprising that cancer, one of the biggest killers in the western world is also closely linked to the RMP. Cancer is often understood as the interplay between the host organism and individual cell regulation (Lobikin et al., 2012), leading to questions on the relative importance of the role played by genetics, epigenetics and the environment. While the mutation centered models of cancer have led the field of research for many years, attention has moved to recognize the importance of the cellular environment. Cancer is fundamentally a developmental disorder of cell regulation, where there is a loss of the organizational capacity of the surrounding environment (Chernet and Levin, 2013b); the RMP is a key element in this environment as the cell membrane is where the cell meets its environment and where it interacts with biomechanical, biochemical, and bioelectrical gradients, all of which impinge the gene regulatory networks. Here we refer to bioelectrics as the EFs that are produced (the spatial and temporal ion flow) and sensed by non-excitable cells. A gateway through the cell membrane exists in the form of multiple ion channels that allow the controlled passage of specific ions. As mentioned earlier, the membrane potential is a key biophysical signal in non-excitable cells that regulates important activities such as proliferation and differentiation and is typically cell type specific (Table 1). Cancer differs from normal cells by the relatively depolarized state of its cells (Cone, 1974; Binggeli and Cameron, 1980; Binggeli and Weinstein, 1986); even as far back as the late 1930s tumors were detected based on their voltmeter readings (Burr et al., 1938; Burr, 1940). The RMP of cancer cells is more depolarized than their normal counterparts; this is illustrated in Figure 2 which shows a series of tumor cells having a RMP that varies from ∼−5 mV to −50 mV (Yang and Brackenbury, 2013). The key hyperpolarizing channels are the Ca^2+^ activated K^+^ channel (KCNMA1), EAG (KCNH1), KV1.3 (KCNA3), KV3.4 (KCNC4), K_ATP_ (KCNJ8/11), and K_2P_ channels (KCNK) whereas depolarisation is mediated by ERG (KCNH2), K_ir_ (KCNJx), and chloride channels (CLCN1 and 2) (Yang and Brackenbury, 2013). This is very similar in range to non-tumor proliferating cells but not quiescent and more fully differentiated cells that are more polarized. The importance of the membrane potential in differentiation can be seen from the experiments of Sundelacruz et al. (2008) and Lange et al. (2011) where forced depolarization maintains cells in a stem-cell like state. Similarly, the depolarization of cells is able to induce a metastatic phenotype. This is particularly interesting as it demonstrates the “cancer at a distance” phenomenon. In *Xenopus* tadpoles, the depolarization of ‘instructor cells’ at the tip of the tail triggers serotonergic signaling that changes the behavior of all melanocytes in the tadpole (Blackiston et al., 2011; Chernet and Levin, 2013a,b). These melanocytes exhibit properties of metastasis such as over-proliferation, cell shape change that facilitates migration, and colonization of other organs and tissues, but the hyperpolarization of cells is able to inhibit oncogene induced tumorigenesis. For example, K_ir_ and constitutively open GlyRF99A, hyperpolarized cells and prevented the formation of tumors despite the strong expression of a co-transfected oncogene (Xrel3) (Chernet and Levin, 2013b); this was confirmed through the use of several different hyperpolarizing channels, indicating that tumor suppression was due to the RMP rather than any one specific channel. Cells derived from cancers with limited therapeutic choices such as those found in triple negative breast cancer patients have been engineered using an L-type voltage-gated Ca^2+^ channel (CaV1) that lacks inactivation. This channel is usually activated by membrane depolarization allowing Ca^2+^ influx; however, the engineered channel is activated at the RMP of −10 mV resulting in an inappropriate influx leading to apoptosis induced cell death (Yu et al., 2017).

Ion channels are good therapeutic targets (see for example, Humphries and Dart, 2015); however, the RMP is influenced by multiple channels and so it is possible that different combinations of ion channel modulating drugs and biologics may be required to effectively change a given RMP. One model of cancer formation is the stem-cell model, where specific cancers arise from stem-cell niches (e.g., basal cell carcinomas of the skin). Changes in the RMP at specific locations appear to act as a source of non-genetic information that affect developmental processes including cancer, and appears to be an untapped treatment mechanism in the war against cancer.

## Cell Migration and Wound Healing

Tissue wounding is an interesting phenomenon because the electrical potential generated by the ion movement in healthy tissue is disrupted and a significant EF is generated that is necessary for wound healing (Reid and Zhao, 2014). Indeed, the EF over-rides other well-accepted physiological cues and initiates directional cell migration into the wounded area. Wound generated EFs are produced by the directional flow of charged ion species. Some of this ion flux will be due to leakage from damaged cells [which themselves have membrane potential dependent repair mechanisms (Luxardi et al., 2014)] and tissue immediately after injury. However, large currents are generated for days after wounding that are not accounted for by immediate injury.

Epithelial wounding has been extensively studied, however, little is reported on the role of the membrane potential in response to wounding and healing. The maintenance of the structural integrity of epithelia is crucial to the function of this tissue type, and healing after injury has been described by two major mechanisms, cell migration and cytoskeleton reorganization (Chifflet et al., 2005). Chifflet et al. (2005) demonstrated that the ENaC ion channel enhanced membrane depolarization occurs at the leading edge of wounds, gradually extending toward neighboring cells, and that this depolarization supports the development of the characteristic actin reorganization found in healing cells. Actin reorganization is evident by the formation of actin cables that form at the leading edge of cells, analogous to the tightening of a purse string as the cells close the wound.

The effects of applied EFs in the wound healing process are becoming apparent (Nuccitelli, 2003). Nuccitelli (2003) speculated that when a cell is placed in an EF, the voltage across the plasma membrane will be modified the most in regions that are perpendicular to the EF lines. The ends of the cell that face the two poles of the field will experience the largest effect. Since current passing through a cell will encounter resistance predominately at the cell membrane, he speculated that a 100 μm long cell in a 100 mV/mm EF will experience a voltage drop of 10 mV, with the plasma membrane facing the positive pole having 5 mV more across it, and the membrane facing the negative pole having 5 mV less. In a cell with a membrane potential of −70mV, voltage-gated ion channels open following a depolarization of ∼10–20 mV, and thus would not be affected by a change of 5 mV. Open channels, however, may result in differential distribution of ions within the cell, with positive ions experiencing a larger force driving them into the cell at the membrane region facing the positive pole of the EF. In cells with membrane potentials that are inherently more depolarized, the effects may be more apparent. Such cells include MSCs and un-differentiated cells that have RMPs below −30mV. Some fully differentiated cells also have more depolarized RMPs, including chondrocytes (Lewis et al., 2011a) and osteoblasts, whereas other cells function with a positive RMP. Broken bones have been reported to heal more efficiently when an EF is applied across the break. Chondrocytes, in callus formation, and osteoblasts both have RMPs below −40 mV, however, it is the MSCs of the periosteum (Zhao et al., 2011; Berendsen et al., 2014) that are believed to migrate to the damaged site under the EF prior to differentiating into these cell types. Thus, in surface wounds or bone damage, a depolarized cell membrane appears to be key in wound healing through epithelial cell and MSC cell migration and cytoskeleton reorganization, respectively. The ion channels underlying these effects remain to be established.

## Pigmentation

Finally, pigmentation in mammals is generally a membrane potential dependent process. In mammals, pigment cells such as skin and uveal melanocytes, and retinal pigment epithelial (RPE) cells are non-excitable cells that contain melanosomes which are lysosome-related organelles that synthesize melanin, the main pigment that colors eyes, skin and hair (Sulem et al., 2007). Melanin is essential for the protection of the skin and eyes against solar ultraviolet (UV) radiation. The changes in intracellular Ca^2+^ regulates the concentration of melanin in pigment cells. These intracellular free Ca^2+^ concentrations are regulated by the membrane potential of the pigment cells. Membrane depolarisation mediated by different ion channels causes the delay of transient potential receptor potential (TRPA1) ion channel inactivation, this leaves the channels open for longer and causes the sustained Ca^2+^ response required for melanogenesis (Bellono et al., 2013). It has been reported that the activation of TPC1 ion channels, which are highly permeable to Ca^2+^ (Lagostena et al., 2017), mediate changes in the membrane potential of the melanosomes, could facilitate the fusion between the melanosomes and other organelles, the plasma membrane, or protein transport vesicles (Cang et al., 2014). This could initiate the melanin transfer in the skin and hence enable protection of the genetic material of keratinocytes against UV radiation damage.

## Conclusion

Over the past several years it has become clear that the RMP is far more widely important to biology than just a firing mechanism for action potentials of excitable cells but rather plays a central role in several biological functions. Modulation of the membrane potential is a potential new target for an additional range of drugs which target a range of diseases and biological functions from cancer through to wound healing and is likely to be key to the development of successful stem cell therapies. The continued exploration of ion channels, which have, in the past been seen as redundant is likely to become increasingly important as these mechanisms are further understood as we seek ever more therapeutic targets.

## Author Contributions

All authors made substantial contributions to the conception or design of the work and interpretation of data for the work, participated equally in drafting the work or revising it critically for important intellectual content, and approved the content for publication and agreed to be accountable for all aspects of the work in ensuring that questions related to the accuracy or integrity of any part of the work are appropriately investigated and resolved.

## Conflict of Interest Statement

The authors declare that the research was conducted in the absence of any commercial or financial relationships that could be construed as a potential conflict of interest.

## Abbreviations

CaV1: L-type voltage-gated Ca^2+^ channel
CNS: central nervous system
E_ca_: equilibrium potential of Ca^2+^ ions
EF: electric field
ENaC: epithelial Na channels
GlyRF99A: glycine-gated chloride channel mutant
IS: intrastrial space
K_2P_: two-pore domain K^+^ channels
K_ATP_: ATP-sensitive K^+^ ion channels
KCNE1: K^+^ voltage-gated channel, subfamily E, regulatory subunit 1
KCNJ10: K^+^ voltage-gated channel, subfamily J, member 10
KCNQ1: K^+^ voltage-gated channel, subfamily Q, member 1
K_ir_: Inwardly rectifying K^+^ channels
MSCs: mesenchymal stem cells
NaV: voltage-gated Na channels
PBMCs: peripheral blood mononuclear cells
PIP_2_: phosphatidylinositol 4,5-bisphosphate
PVN: paraventricular nucleus
RMP: resting membrane potential
RPE: retinal pigment epithelial cells
SCN: superior chiasmatic nucleus
SK: small-conductance Ca^2+^-activated K^+^ channel
TPC1: two pore segment channel 1
TRP: transient receptor potential channels
TRPA1: transient receptor potential cation channel, subfamily A, member 1
TRPV4: transient receptor potential cation channel, subfamily V, member 4
TRPV5: transient receptor potential cation channel, subfamily V, member 5
VSM: vascular smooth muscle.
